# Experiences of primary care physicians managing postpartum care: a qualitative research study

**DOI:** 10.1186/s12875-021-01494-w

**Published:** 2021-06-30

**Authors:** Zhimin Poon, Esther Cui Wei Lee, Li Ping Ang, Ngiap Chuan Tan

**Affiliations:** 1 SingHealth Polyclinics, 167, Jalan Bukit Merah, Connection One, Tower 5, #15-10, Singapore, 150167 Singapore; 2 SingHealth-Duke NUS Family Medicine Academic Clinical Program, Singapore, Singapore

**Keywords:** Primary care physician, Postpartum care, Mother, Healthcare system

## Abstract

**Background:**

The postpartum period is redefined as 12 weeks following childbirth. Primary care physicians (PCP) often manage postpartum women in the community after uneventful childbirths. Postpartum care significantly impacts on the maternal and neonatal physical and mental health. However, evidence has revealed unmet needs in postpartum maternal care.

**Aim:**

The study aimed to explore the experiences of PCPs in managing postpartum mothers.

**Methods:**

Four focus group discussions and eleven in-depth interviews with twenty-nine PCPs were conducted in this qualitative research study in urban Singapore. PCPs of both gender and variable postgraduate training background were purposively enrolled. Audited transcripts were independently coded by two investigators. Thematic content analysis was performed using the codes to identify issues in the “clinician”, “mother”, “postpartum care” and “healthcare system & policy” domains stipulated in “The Generalists’ Wheel of Knowledge, Understanding and Inquiry” framework.

**Findings:**

PCPs’ personal attributes such as gender and knowledge influenced their postpartum care delivery. Prior training, child caring experience and access to resource materials contributed to their information mastery of postpartum care. Their professional relationship with local multi-ethic and multi-lingual Asian mothers was impacted by their mutual communication, language compatibility and understanding of local confinement practices. Consultation time constraint, awareness of community postnatal services and inadequate handover of care from the specialists hindered PCPs in the healthcare system.

**Discussion:**

Personal, maternal and healthcare system barriers currently prevent PCPs from delivering optimal postpartum care.

**Conclusion:**

Interventions to overcome the barriers to improve postpartum care will likely be multi-faceted across domains discussed.

**Supplementary Information:**

The online version contains supplementary material available at 10.1186/s12875-021-01494-w.

## Statement of significance

### Problem or issues

Women often have unmet maternal needs in the postpartum period.

### What is already known

Most postpartum mothers return to their community after uneventful childbirth, and are ideally managed by Primary Care Physicians (PCPs). Yet evidence has shown suboptimal postpartum care for mothers in the community.

### What this paper adds

PCPs face personal, maternal and healthcare system related barriers when they manage postpartum mothers. Multipronged approach will need to be adopted to enhance the enablers and improve PCPs’ practices in postpartum care.

## Introduction

Postpartum period for a mother has been re-defined from a time frame of 6 weeks after the birth of her child to a postnatal period of 12 weeks [1]. The extension lengthens the window period to optimize the medical care of the postpartum mothers and their neonates. The WHO guidelines recommend comprehensive assessment of both women and child during this postpartum period. The postpartum visit should ideally cover the evaluation of the physical, psychological, and social health of the postpartum women, their breastfeeding practices, nutrition advice, family planning, and development and growth of the child [2].

Postpartum period is a critical time to support these mothers and their new-born child as they often encounter major unmet needs [3]. In the early postpartum period, especially for mothers with early hospital discharges, they are unsure of the healthcare services to address their breastfeeding difficulties [4]. They perceive lack of mental health screening and support [3] during this period, which is a stark contrast to the care they receive during the antenatal period [5]. Even at the later postpartum period, mothers still face persistent breastfeeding problems and inadequacy in knowledge and skills in providing care for the child [6]. In a national survey conducted in the United States, 90% of mothers completed their postpartum visit. However, 42% of them reported that key health topics such as birth control methods, postpartum depression, healthy eating, appropriate types of exercise, changes in sexual responses and feelings were discussed with their primary care physicians (PCPs) during only 48–60% of their consultations [7].

Singapore is a developed urbanised island-state without national clinical practice guidelines on postpartum care. Approximately 39,000 local multi-ethnic Asian women require postpartum care annually [8]. Their antenatal care is largely provided by the obstetricians in tertiary care. Women with no intrapartum complications are discharged from hospitals within 24 to 72 h after childbirth [9]. Upon discharge, they are usually given a 6-week postpartum review with their obstetrician or advised to self-arrange a review with their PCP. Unlike other healthcare systems, midwifery has mostly ceased in the local primary care setting [10].

Only 38.4% of Singapore residents have a regular family physician [10]. Nonetheless, PCPs are much more easily accessible as they are located within the community and same-day consultation appointments are often available as needed. As most women return to the community within a few days after childbirth, primary care is ideally placed to offer comprehensive, continuous, and coordinated care to postpartum mothers and their infants [11]. Local PCPs are trained in both maternal and child health and have several opportunities to interact with them. These include interactions with the mother during their newborns’ neonatal jaundice follow-ups, developmental and growth assessments, childhood immunisations in the early postpartum period and at the 6-week postpartum review.

However, literature suggests gaps in postpartum service in primary care. A systemic review shows variability in the existing international clinical practice guidelines on postpartum care in community setting [11]. The paucity and inconsistent recommendations result in variability and quality of post-partum care provision among PCPs. In addition, their roles, capacity, and capability in delivering postpartum care are little understood. A local study has revealed unmet personal needs of postpartum mothers. Ong SF et al. have highlighted the lack of clinician support and advice to primiparous mothers who experienced difficulty in achieving exclusive breastfeeding [12]. The local fee-for-service healthcare system does not assign them to a personal PCP [9]. Some of these mothers had difficulty accessing a healthcare provider after discharge and felt apprehensive in contacting a health care provider for issues deemed “non-medical” such as infant care skills.

This qualitative research study aimed to explore the experiences of PCPs in their postpartum care delivery to Asian mothers throughout the revised extended postpartum period in an urbanised society. Specifically, it aimed to identify the factors relating to their personal attributes and training, perceived roles and interaction with postpartum mothers and issues they encountered with the healthcare system in delivering postpartum care.

## Methods

### Study design

The study employed qualitative research method in which focused group discussions (FGD) and in-depth interviews (IDI) were organised to gather PCPs’ perspectives. FGDs were organised to gather the diversity of the views when PCPs interacted with each other during their group discussions. The flow and content of the interview guide was revised with each FGD. IDIs were then conducted to clarify the themes which emerged during the analysis.

### Conceptual framework

“The Generalists’ Wheel of Knowledge, Understanding and Inquiry” was selected as the conceptual framework to design the topic guide [13]. The framework underpins the generation of new knowledge for Family Medicine via research, which is apt for this study, as it seeks to inquire and understand the practice of PCPs in postpartum care. It encompasses the major domains which potentially influence the PCPs in postpartum care. The framework covers issues pertaining to the “clinician”, “mother”, “postpartum care” and “healthcare system” domains, and inter-domain issues such as “information mastery” on the ways in which PCPs acquire knowledge on postpartum care.

### Site

The FGDs and IDIs were conducted at a public primary care clinic that serves an estate populated with young families in northeast Singapore [14]. Subsequent gathering of qualitative data was carried out over online teleconferencing platform, such as Zoom® during the lock-down amidst the COVID-19 pandemic in Singapore.

### Period of study

The study was conducted between February to October 2020.

### Researcher characteristics

The study team comprised of one male and two female PCPs and a female nurse manager practicing in SingHealth Polyclinic, a public primary care instituition in Singapore. The females in the study team have all experienced childbirth and received postpartum care in Singapore.

### Study population

The target participants were PCPs in both the public and private general practices in urbanised community setting. They had to be actively practising (defined as 24 clinical hours a week) and self-declared to be involved in the provision of postpartum care.

### Recruitment

PCPs were invited either in person or via emails to participate in the study. The invitations included the reasons for doing the study and emphasized that participation was voluntary. Purposive sampling [15] from the professional networks of the study team was conducted to include PCPs of different ages, gender, ethnicity, medical training and location of practice. Their demographic profiles, background Family Medicine training and types of primary care practices were postulated to influence their postpartum care practices. Informed consent was taken prior to commence of the interviews.

### Interviews and transcribing

The participants completed a brief demographic data questionnaire before taking part in either an FGD or IDI via face to face interactions or remotely using an online teleconferencing platform. The PCPs were provided with a topic guide (Supplementary File 1) prior to the commencement of the interviews for their awareness and preparedness. The moderator for both FGDs and IDIs was PZM, a female PCP, with approximately 10 years of clinical experience in family medicine and Master of Medicine postgraduate family medicine qualification. Another co-investigator, either ELCW or ALP assisted PZM during some of the interviews to take field notes. The IDIs and FGDs were conducted in quiet rooms at the study site. All participants were reimbursed with grocery store vouchers of SGD20 (estimated USD15) as tokens of appreciation for their time spent at the sessions (45 to 90 min). The interviews were audio-recorded and transcribed verbatim by professional transcriber. Next, the principal investigator audited and rectified the transcripts to ensure accuracy.

### Coding

Data collection and analysis occurred concurrently. The transcripts were read in full by two investigators, who then coded independently to derive a first coding frame. Regular meetings were held to discuss and modify the coding frame for data analysis. Repeated and common codes identified during these discussions between the investigators were crosschecked and clarified during subsequent FGDs or IDIs with participants. The mutually agreed codes were assembled into a coding frame. Any difference or disagreement in coding was deliberated between the investigators to reach a consensus. Eventually, the codes from the first six interviews were used to finalise the coding frame, which was then deployed to code the remaining 9 interviews. Data saturation was defined as the point when no new code emerged [16]. The codes were then used to identify emerging themes, which were then categorized according to the four key domains in the Generalist Wheel theoretical framework.

NVivo software version 12 was used to manage the transcripts and coding and to facilitate the final data analysis.

## Results

A total of 31 PCPs were approached and 29 of them agreed and participated in 4 FGDs and 11 IDIs. The participants included 5 General Practitioners (GPs) in private practice and 24 PCPs in various public primary care clinics (polyclinics). Their ages ranged from 26 to 61 years. The FGD or IDIs lasted between 53 to 87 min, averaging 69 min. Their characteristics are represented in Table 1.

**Table 1:** Demographic characteristics of study participants (*N* = 29)

**Characteristics**	**N (%)**
*Age (years)*	
25–34	16 (55.2%)
35–44	7 (24.1%)
> = 45	6 (20.7%)
*Ethnicity*	
Chinese	24 (82.8%)
Malay	1 (3.4%)
Indian	1 (3.4%)
Other	3 (10.4%)
*Postgraduate training*	
MBBS	9 (31.0%)
Graduate Diploma in Family Medicine	4 (13.8%)
Master of Medicine (Family Medicine)	11 (37.9%)
Fellow of the College of Family Physicians, Singapore	5 (17.3%)
*Designation*	
Medical Officer^a^	4 (13.8%)
Resident Physician^b^	4 (13.8%)
Family Physician^c^	16 (55.2%)
General Practitioner^d^	5 (17.2%)
*Site of practice*	
Public healthcare institutions	24 (82.8%)
Private GP practices	5 (17.2%)

^a^Qualified physicians granted conditional or full medical registration by the Singapore Medical Council (SMC) without postgraduate qualification

^b^Qualified physicians with at least an undergraduate medical degree, granted full medical registration and with at least 3 years of clinical experience

^c^Registered medical practitioners with relevant and recognized postgraduate qualifications (Graduate Diploma in Family Medicine, or a Masters in Family Medicine) and with at least 3 years clinical experience

^d^Registered medical practitioners with relevant and recognized postgraduate qualifications (Graduate Diploma in Family Medicine or a Masters in Family Medicine) working in the private setting

The results are grouped into 4 main domains based on the conceptual framework. They include the PCP’s personal attributes towards postpartum care; their information mastery on postpartum care; professional relationship with postpartum mothers; and their interaction with the healthcare system and policies. All themes and subthemes are supported with corresponding verbatim.

### Role of the PCP in postpartum care

#### Early point of contact in the postpartum period

The PCPs concurred that they were often the first touchpoints for postpartum mothers. The location of their clinical practices within the estates in close proximity to the mothers’ residences and availability of same-day medical appointment make PCPs easily accessible in Singapore.

*“…we are the first point of contact for most of them…if they have any problems postpartum, they will usually go to the nearest polyclinic or GP (General Practitioner) to settle some of their queries.”* (PC20, Male GP)

#### Scope of postpartum care

PCPs indicated that the care they provide following childbirth is not limited to the immediate postpartum period but encompasses a larger scope including providing longer-term medical and mental health support for both mother and child. In the early postpartum period, medical needs such as episiotomy or caesarean wound care, gestational diabetes or pregnancy induced hypertension can be reviewed by PCPs. Screening the mental health needs of postpartum women and provision of breastfeeding support are other important postpartum assessments that can be fulfilled by PCPs.

*“…we are more familiar with the mental health aspect and we are the ones that are mainly seeing them after they have delivered for a much longer time if they have issues.”* (PC24, Female Family Physician in Polyclinic)

Subsequently, education and preventive care including family planning and cervical cancer screening can also be discussed with the mother.

*“…giving them advice about contraception and a follow up PAP smear.”* (PC11, Female Resident Physician in Polyclinic)

#### Coordination of care

Most PCPs viewed themselves as coordinators of care, who can identify areas of need of postpartum mothers and match them up with available resources in the community. This can be made possible if they have adequate support such as engaging nurses and midwives to help with holistic assessment of the mother and tie-ups with community resources.

*“The doctor, before even seeing the patient, will already have a very clear understanding of what are the issues that were identified by the nurse for this patient, and then, will be able to conduct a streamlined type of consultation, then refer the patient on to the necessary services that may be available. They may even think about engaging some form of community services, so that the doctor can also refer out to community services.”* (PC1, Male Family Physician in Polyclinic)

#### Involvement in antenatal care

Some PCPs strongly believed in the need for their involvement in the antenatal care of a pregnant woman. This early establishment of physician-mother interaction can contribute to rapport building and potentially improve eventual long-term care for the mother.

*“I think it becomes more natural if we are actually doing antenatal care, we establish rapport and we look after the prospective mummy, and then at a certain time, we refer in for delivery. It becomes very natural for the lady to come back.”* (PC14, Male Family Physician in Polyclinic)

### Personal attributes towards postpartum care

#### Gender

The PCPs identified the gender of the physician to be a significant factor influencing the consultation agenda with a postpartum mother. As postpartum care involved examination of the more private areas, such as the genitalia and the breasts, male PCPs reflected that they were less likely to be consulted by postpartum women. The latter might not bring up the related issues even if they were attended by male PCPs.

*“Some of them, they prefer a female doctor instead of a male doctor, especially if it involves a lot of checking (of) the breast and also the private part(s)” (PC6, Male Medical Officer in Polyclinic)*

*“My credibility is definitely less than hers. She (female colleague) breastfed four children of her own. I can’t breastfeed any of my children. I have no credential.”* (PC25, Male GP)

#### Background knowledge and capability in postpartum care

PCPs reported a spectrum of personal knowledge on postpartum care. They attributed their variable experience and immersion in postpartum care to their prior medical training. They would readily seek advice from their peers or consult specialists in postpartum care. The PCPs either called the obstetricians whom they were acquainted by phone, or consulted them via formal referral.

*“We do have a doctors’ WhatsApp chat(group) for the doctors under that clinic. So (I) do ask my peers and my colleagues for advice.”* (PC23, Female GP)

*“I have… three classmates who are obstetricians, whom I can (contact) …are just a text away.” (PC25, Male GP)*

*“well, as primary care physicians, …. many times, you can make a referral” (PC12, Male Family Physician in Polyclinic)*

#### Personal child-caring experience

PCPs, underpinned by their own personal experience, were more confident in advising breastfeeding and parent crafting to postpartum mothers. The experience stemmed from their personal roles as mothers and fathers who were actively involved in the care of their own children. They could empathise with the difficulties faced by the postpartum mothers.

*“I think there’s a difference before and after becoming a mother myself…after experiencing (it) yourself as a patient, then it gives me more confidence.”* (PC4, Female Family Physician in Polyclinic)

*“It depends on how hands-on I am as a father. So, as a very hands-on person, I don't feel disadvantaged, as opposed to someone who’s not had a child before”* (PC13, Male Family Physician in Polyclinic)

In contrast, single and married physicians without children had less confidence in addressing these issues. They tended to engage the help of more knowledgeable people around them or were more inclined to refer the mothers to other providers.

*“I don’t have any personal experience myself, so if they (mothers) do have issues about latching or how to deal with engorgement, I don’t really know how to advise them… If I examine them and there’s mastitis, I can treat, but with issues to prevent that from happening … I’m really not very well-equipped, so I will ask them to either call the lactation consultant.”* (PC11, Female Resident Physician in Polyclinic)

### Information mastery on postpartum care

#### Medical training on care of well-women and well-child

Most PCPs claimed to know the basic clinical examination of a postpartum woman. They were aware on the assessment of lochia cessation, wound recovery, pain control, screening for post-natal depression, breastfeeding, and preventive care, such as cervical cancer screening and contraception advice.

Some PCPs reported paucity of related formal training in postpartum care. Those who self-declared to be deficient in postpartum care, indicated their intent to embark on self-learning.

*“…I also agree that I don’t recall any structured teaching on postpartum care, but for me, it was also mostly likely self-learning. A bit of teaching from likely, maybe the O&G (Obstetrics and Gynaecology) and Paediatric posting, but VERY, VERY MINIMAL, but I knew that it would be something I might see in primary care, so then, you know, I had to read myself.”* (PC5, Male Resident Physician in Polyclinic)

Whilst most PCPs concurred that medical undergraduate and postgraduate training in family medicine equipped them with the medical knowledge and skills in identifying abnormal and dangerous presentations in postpartum mothers, they were not trained in assessing breastfeeding and normal feeding behaviour of the baby.

*“…Knowledge wise, I think most of us are not actually well trained in that aspect as in, how to advise them on, … nutrition, how to take care of their own mental, physical health. And also, not just taking care of them,, but taking care of neonates also is important, … because most of the ladies who come in at this time, they have a lot of questions about their baby as well, which also affect their own emotional, their mental health.”* (PC20, Female Family Physician in Polyclinic)

#### Access to resource materials

Most PCPs highlighted the lack of local primary care postpartum care guidelines, which they attributed to their variable scope of practice. Most relied heavily on their own informal resources to update their postpartum care knowledge, assist their consult process and to teach the more junior physicians within their institutions. They suggested references to web-based education materials from a local tertiary women and children hospital, previous training courses and resource persons within their clinic practice.

*“(In) the SHP (SingHealth Polyclinics) doctor guidebook, there’s a postpartum guide over there. And then, there’s some helplines you can call, like the lactation services that are available in KKH (Women’s & Children’s) Hospital. And also, there’s online learning module about breastfeeding. So for those doctors who are not so familiar, maybe it’s something that they tap on to.”* (PC4, Female Family Physician in Polyclinic)

*“I did consult some of the more senior doctors, who also provided me with … some resources. The online lactation services (referring to KKH), they do have their own resources that you just download and refer. Overtime, … you just learn from there. When juniors ask you, you just refer them to the similar resources.”* (PC20, Female Family Physician in Polyclinic)

*“…is to look at the latest update GDFM (Graduate Diploma in Family Medicine) notes. Because I’m a faculty member, I have access. Number two, will be open resource material over the internet. Number three, is paid resources, likely UpToDate. Number 4, I have informal resources, likely my co-locating obstetrician and gynaecologist.”* (PC25, Male GP)

### Professional relationship with postpartum mothers

#### Awareness and de-conflicting postpartum mothers’ confinement practices

In the early postpartum period, traditional confinement practices among the local multi-ethnic Asian mothers are prevalent, despite a majority of them receiving western education in Singapore. Such practices may not be evidence-based but can potentially interfere with the PCPs’ efforts in addressing pertinent postpartum needs such as lactation and skin ailments.

*“From my experience, confinement nannies are universally wrong in their breastfeeding advice. Usually, they will supplement with the formula, which is not what we are taught, but you are supposed to clear the breast and keep latching until the supply comes, but if you introduce supplement, you just compromise the whole thing.”* (PC12 Male Family Physician in Polyclinic)

*“.. some of them (mothers) don’t bathe, and then on top of that, they have to wear a long-sleeved pyjamas, and then no air-con, (they) only can use fan in this current (tropical) climate, I think that they can actually get quite a fair bit of eczema.”* (PC22, Male GP)

The PCPs could readily identify myths in confinement practices. However, some of them were less inclined to debunk such practices to avoid adversely affecting their relationship with the postpartum mother, her family or confinement nanny. Therefore, if the PCPs perceived that the confinement practice was harmless, they would not take further action.

*“I see less of a problem now. Of course, there’s still some traditional (confinement practices) … but … as long as it’s not a hindrance to medical care, I think some of these old wives’ tale can be left alone. It doesn’t really matter!”* (PC27 Female Family Physician in Polyclinic)

#### Language compatibility in physician-mother communication

In view of the local cosmopolitan population with a minority of foreign women marrying local men, not all postpartum mothers are proficient in English. Language incompatibility appears to affect mutual communication between the PCP and postpartum mother, even when the spouse or family are available to translate. PCPs felt that important information might be lost during the translation process.

*“…the wife is telling him a lot of things, and … just a few phrases coming out from the husband’s mouth. It’s likely the husband is already filtering whatever needs to be said … I can’t, I can’t “unfilter” (decipher) it myself!”* (PC24 Female Family Physician from Polyclinic)

*“The moment I have an interpreter, … it’s an added barrier. It’s harder. It makes the job double hard.”* (PC25, Male GP)

#### Alternative formal and informal healthcare provider and perceived missed opportunities

The mother-PCP relationship is not steadfast during the postpartum period. PCPs reported that mothers tended to rely on alternative healthcare providers such as their obstetricians, and other readily accessible sources of information from their family, confinement lady and friends to address their postpartum needs.

*“…I think some women, for example, they don’t see the need to tell the primary care physician about the issues they are going through postpartum. Some of them feel that it’s better for them to go to a gynae (cologist) or they might feel that the polyclinic is not the right setting to do it.”* (PC5, Male Resident Physician in Polyclinic)

*“it’s probably due to the community whereby we have a lot of support. For example, … they get the opinion and advice from the confinement lady. Some of them, they actually get those opinion or advice from their parents as well.”* (PC6, Male Resident Physician in Polyclinic)

The PCPs postulated that the postpartum mothers were unaware of the values and purpose of a routine postpartum review and were unfamiliar with the postpartum services available in primary care. This could result in their missed opportunities to optimize postpartum care.

*“.. actually even if they (mothers) feel well, they can be counselled on … contraception that they tend to just gloss over, … postpartum depression when they wouldn’t reach out in the first place. So there are things to screen, but whether they think they need visits, the doctor’s visit, it’s questionable.”* (PC15, Female Family Physician in Polyclinic)

### Challenges relating to the healthcare system and policies

#### Limited consultation time and ineffectual counter-measure

One common barrier pertaining to the comprehensive postpartum reviews highlighted by the PCPs was the inadequate consultation time allocated for a postpartum visit in primary care setting. Most felt that a more realistic time per consult should last from 15 to 20 min, compared to the current 5 to 10 min in real-time clinical practice. The limited time for consult often reduced the comprehensiveness and scope of postpartum issues covered within the postpartum visit. Consequently, the PCPs tended to avoid time-consuming tasks such as mental health assessment or discussing contraception options with the mothers. To overcome the short consult, some PCPs advised mothers to return for a separate visit to tie up loose ends. However, the PCPS recognised that default rate would be high, resulting in incomplete resolution of issues faced by postpartum mothers.

*“It’s actually time that is given to us. If these patients turn up in general pool (referring to walk-in consultation), …, within 6-min consult, it’s very, very challenging to address (the) so many aspects of the maternal health”* (PC2, Female Family Physician in Polyclinic)

#### Awareness of and access to community postnatal care services

PCPs regarded breastfeeding difficulties as a common problem experienced by postpartum mothers. Some PCPs perceived deficiency in managing women with lactation issues. However, they highlighted the inadequate lactation support services in the community. While lactation consultants are available in public tertiary institution, the long wait time for referrals is a hurdle. For those who are cognizant of private lactation consultants, engaging the latter is costly, hindering its access to mothers across different socioeconomic groups in the local fee-for-service healthcare system.

*“We call the lactation consultant (in KKH), but the appointment was very long. So we got the private consultant to come down to help us. And it’s only because we are resourceful enough, that we … look for all these (services), but again, people out there may not be so resourceful, they may not be able to find all these (services)…”* (PC13, Male Family Physician from Polyclinic)

Similarly, PCPs perceived limited access to community psychological services for mothers who were diagnosed with postnatal depression. Many PCPs felt that increasing access to such services would enable them to better manage the emotional needs of some postpartum mothers.

*“It may not be clinical psychologist, but there are counsellors outside who can actually help this lady, so what we need is to have the network of these services. Some of them are in Family Service Centres and … for us to be able to refer, and (for mothers) to reach out to them while they are at home.”* (PC14, Male Family Physician in Polyclinic)

#### Primary-tertiary care interface: handing over of care from specialists

Poor transfer of care from the specialists to PCP impedes transition and continuity of postpartum care of the mothers after their delivery. When postpartum women are discharged after an uneventful pregnancy and short hospital stay, they are given a memo to bring to their PCP for postpartum care. However, the appointment is usually not fixed to any specific PCP. Consequently, the mothers often miss their postpartum review within the stipulated period due to competing demands of their attention. Across public and private sectors, PCPs often do not receive any memos from the obstetricians and rely on the national electronic health records (NEHR) to access information on the mothers’ antenatal and intrapartum history. Furthermore, not all PCPs register with the NEHR agency to access the medical records, leading to fragmented information for postpartum care.

*“Definitely it’s fragmented. There’s actually no proper structure to it… the hospital doesn’t know if the patient comes and we don't know whether how many patients are coming to us. There’s private sector and there’re different (systems in) restructured hospitals, polyclinics, so it’s very fragmented indeed.”* (PC4, Female Family Physician in Polyclinic)

*“It’s definitely not adequate, because some patients (are) follow up private, some patients in government hospitals, and we have SO MANY polyclinics, private GPs, so they choose ANYONE to follow up. The only communication we have is … the memo when the patient brings in. So if the patient NEVER bring(s) in the memo, we will never follow up with them.”* (PC10, Female Resident Physician in Polyclinic)

In the current model of care, a woman is immediately referred to an obstetrician upon diagnosis of pregnancy. The PCPs alluded that this early referral compromises on the rapport with the postpartum mother. This broken linkage results in failure of the PCPs to connect with these mothers after their delivery.

*“Primary care physicians should be the one handling the antenatal care part until the delivery for a normal, uncomplicated delivery. Then the patient will have trust in you and (will) be willing to follow up subsequently AFTER their deliveries. Instead, once diagnosing them as pregnant, we refer them onwards. Is this really what we want in our primary care setting?”* (PC16, Male Family Physician in Polyclinic)

### The way forward: suggestions to strengthen the enablers

#### Team-based practice

PCPs proposed measures to overcome their perceived barriers. Some of them, especially those from the polyclinics, suggested team-based practice to overcome gender and variable levels of competency. The proposed team comprises a mix of different physicians, midwives and nurses. It enables senior experienced doctors to mentor the junior physicians within the practice. It also allows mothers to engage female PCPs in the team to deal with their gender-sensitive issues and private anatomical examination. Task substitution to the midwives and nurses is another suggestion. They can be trained to be lactation consultants to manage breastfeeding issues. The multidisciplinary team can potentially reduce the PCP’s consult time, without compromising on a comprehensive postpartum review of the mothers. Nonetheless such a care model is feasible in polyclinics with a larger pool of primary healthcare professionals, compared to singleton or small GP practices with limited human resources.

*“It may not need a doctor to provide the care. If you look at the previous maternal-child health model in the past, where usually the midwife is the one who provide(s) the postpartum care and to identify what are the potential issue(s) that can arise from the mother. We can train our nurses to do that. If they pick up any issues, then they refer to the doctor as the second line provider.”* (PC2, Family Physician in Polyclinic)

#### Reorganization of antenatal and postpartum services

The PCPs suggested reorganizing antenatal and postpartum services to prepare the mothers for postnatal care. Such antenatal classes in primary care setting aim to familiarise the mothers to the PCPs and induct them on the purpose and importance of postpartum reviews.

*“We need to make the public more aware that such resources are available (in primary care). Antenatally, drum in advice on possible issues that can come up in the postpartum period.”* (PC11, Female Medical Officer in Polyclinic)

*“The people must be trained, the nurses and doctors, to be able to deliver the care when patients turn up…It may not be enough just to read the doctor’s guidebook. There may be a need for some refresher, sort of CME (Continuing Medical Education).”* (PC14, Male Family Physician in Polyclinic)

Clinical practice guidelines were deemed useful by PCPs. Some of them recommended the introduction of guidelines to ensure appropriate handling over of high-risk cases from obstetricians to PCPs and guide the PCPs to manage the well women and well child.

*“If there is a clinical guideline…that would be very helpful.”* (PC25, Male GP)

#### Improve access to ancillary postnatal care services

The PCPs recommended direct access to trained personnel within the clinic or to be able to link to a network of community resources to support postpartum care. This will value add to the PCPs’ services, without the extra step of referring mothers to other postpartum providers and incurring additional cost.

*“If we can have a nurse who has worked in a O&G setting, and who can run parent-craft classes or give proper advice…with regard to milk intake for, not just breastfeeding, but for women who have been giving formula (to their children) …that will be good for parents.”* (PC23, Female GP)

## Discussion

The study explored the issues faced by PCPs with variable postgraduate training background in both the public and private sectors. The findings reveal a spectrum of barriers and enablers which influences postpartum care in an urbanised Asian community. The use of the Generalist Wheel of Knowledge, Understanding and Inquiry as the theoretical framework in this study allows the barriers and enablers to be grouped into key domains. They include the PCPs’ personal attributes towards postpartum care, their information mastery on postpartum care, their professional relationship with postpartum mothers and their interaction with the healthcare system and policies.

PCPs perceived male gender as a major barrier due to certain aspects of postpartum care requiring examinations of more intimate areas. Preference for a gender concordant physician in performing sensitive physical examinations were reflective of inclinations of only a fraction of female patients in other studies [17, 18]. Other more important factors considered by them when selecting a provider include experience and clinical competence, which were reflective of PCPs’ perceived enablers for addressing postpartum care needs in this study. A systematic review has shown that women with preferences for female physicians felt that the latter provided more patient-centred communication [19]. Asking patients for permission to perform an examination, explaining why the examination is important and providing female clinic assistant to be present during the examination are all potential strategies that can be explored, especially among the male PCPs.

The lack of formal structured training, self-efficacy in accessing informal training and resource materials influenced PCPs’ mastery of postpartum care. In developed countries such as Canada [20], Norway [21] and Australia [22], PCPs similarly reported that they were aware of the benefits of breastfeeding but lacked basic knowledge to assess and advise on breastfeeding. Postpartum care training, including assessing breastfeeding and normal feeding behaviour of the baby can be incorporated into the national Family Medicine training programmes to increase the competency of PCPs in this field [23].

A systematic review on the information seeking behaviour of physicians showed the most common sources for information included text sources, asking colleagues and electronic databases [24]. Convenience to access, habit, reliability, speed of use, high quality and applicability made information seeking more likely to occur. Transdisciplinary collaboration between PCPs and specialists should be forged to develop clinical practice guidelines which are culturally-adapted for local PCPs and postpartum mothers. These guidelines provide easily accessible and reliable contextual evidence-based recommendations to standardize postpartum care to reduce unnecessary variations in primary care practices, be it public or private sector [25]. Asking colleagues is a common behaviour of information seeking among primary care physicians [26]. While access to a colleague physically may be challenging for a solo-practice general practitioner, instant messaging apps have been shown to be a promising system as a means of communication between health care professionals [27]. Although the use of instant messaging apps can over the physical barrier of accessing a colleague, several ethical and user issues have to be taken into consideration such as confidentiality and privacy, proper record keeping and data storage, consent from parties involved and authentication of users [28].

In their interaction with the postpartum mothers, language incompatibility can sometimes be a barrier in effective communication. Jaegar et al. reported that language incompatibility is commonly experienced by PCPs in Switzerland, a country with significant migrant population [29], akin to that in Singapore. Language barriers can potentially threaten patient safety during consultation on diagnosis, management risks and advices [30]. PCPs may encounter more cases of physician–patient language incompatibility due to the increasingly multinational population in Singapore. While group practices allow the physician to tap on fellow physicians’ bilingual capabilities, single practice GPs may not be well supported and use of professional interpreters is practically non-existent in local medical practices. An option is for these GPs to strategically hire clinic assistants with various language proficiencies to serve as ad-hoc translators.

A review on the Asian traditional beliefs and practices in the postpartum period showed that traditional postpartum practices persist in many Asian cultures [31]. Failure to adhere to such confinement practices is widely believed to result in long-lasting health consequence for the mother and child. PCPs avoided advising postpartum mothers against some of these practices, which were deemed as harmless, in order to preserve good doctor-patient relationship. Nonetheless, they should be cognizant of such traditional beliefs and practices to trigger discussions with the mothers. Such deliberation provides opportunities for the PCPs to clarify the safety of these practices and to give timely appropriate advice to the mothers and their families.

The limited time for consult is a prevalent concern of the PCPs on the healthcare system. Australian GPs had reported that postpartum consultations usually lasted longer than 15 min, with some even extending to more than 25 min [32]. Mothers similarly reported lower satisfaction with rushed medical consultations and were less likely to discuss their postpartum matter [33, 34]. Setting up dedicated appointment-based maternal-child clinic which caters for longer consult time may potentially address this issue. The proposed bundled care for mother and child necessitates a different healthcare financing system to allow adequate remuneration for the additional time needed in these dyad consultations. Currently, local primary healthcare providers rely on the fee-for-service to cater to the needs of the majority of their patients. The cost of consultation may deter mothers from adhering to their postpartum reviews. Allowing them to utilise their personal or their spouses’ mandatory national health saving fund (Medisave) should be considered an option to alleviate their out-of-pocket consultation payment [35].

The PCPs have suggested team-based care to address multiple barriers faced by postpartum mothers. Clinician diversity in a team allows postpartum mothers to select their preferred PCPs to discuss gender-specific issues. The nurses in the team can also be trained to be midwives and lactation consultants. Local primary care institutions have set up team-based collaborative care for other services such as memory and cognition clinic [36] for subset of the patient population. This care model can be extended to postpartum services. The polyclinics with larger and more diverse pool of in-house healthcare professionals have the capacity to support team-based care for mother and child. The local primary care network (PCN) scheme [37] creates opportunities for private GP to organise themselves in similar team-based care model, leveraging on the economy of scale to optimize human resources. Under such a scheme, postpartum mothers can receive care through a multi-disciplinary team supported by PCPs, nurse counsellors and primary care coordinators.

Competing demand for attention from their child by mothers and perceived poor accessibility to postpartum support services are cited by the PCPs as other barriers in this study. The advent of telemedicine, especially amidst the current Covid-19 pandemic, potentially provides an alternative portal for postpartum mothers to seek medical attention at the convenience and comfort in their residence. Telehealth services have been shown to cater to the needs of postpartum mothers. A systematic review on telehealth interventions used to improve obstetrics and gynaecology outcomes showed that early postpartum weight loss and breastfeeding exclusivity can be facilitated by telehealth [38]. Besides using text communications and Web-based platforms focused on breastfeeding to improve rates on breastfeeding exclusivity, Brown et al. suggested that telepsychiatry can be used to screen and follow up for postpartum depression [39]. A systematic review on the effectiveness of telemedicine interventions to address maternal depression reported improved clinical outcomes via telemedicine in the majority of the studies [40]. The limited telehealth services currently available in local public and selected private primary care clinics serve patients with stable long-term diseases such as hypertension or acute medical conditions such as upper respiratory tract infection. The feasibility of its extension to postpartum care remains unknown and awaits further health service research evaluation.

### Strengths and limitations

The use of the Generalist Wheel of Knowledge, Understanding and Inquiry to explore the challenges faced by PCPs in providing postpartum care is novel. It seems to be an ideal framework to seek understanding of the issues not only in the PCP domain but also focus on its relationship with the mothers, impact from the healthcare system and policy and their mastery of the postpartum care practices. The model provides ease of reference to the multitude of issues across domains and the understanding that complex intervention that addresses the inter-linked obstacles is likely to be necessary to optimize success in improving postpartum care for mothers.

This study has its limitations. Qualitative research is exploratory in nature and therefore the results of this study may not be generalizable to the population of local PCPs. However, the breadth and depth of the issues are possibly maximised via purposive recruitment of PCPs and GPs to identify a wide range of issues across the different primary care providers. Secondly, the findings are not quantifiable to allow prioritization in the design of complex intervention to address the issues. A qualitative research study to gather the views of the mothers on their postpartum care and a questionnaire survey to quantify the barriers and enablers will be implemented in the next phase of the study.

## Conclusion

This study reveals multiple barriers relating to physician, maternal and healthcare systems and identifies facilitators which potentially enable PCPs to improve their provision of postpartum care. The results allude to the possibility of a multipronged intervention to address the barriers, so that PCPs can deliver quality postpartum care. Further research involving postpartum mothers and a survey to measure the magnitude of the barriers will triangulate with the findings in this study.

## Supplementary Information

**Additional file 1.** Interview Guide.

## Data Availability

The datasets analysed during the study are available from the corresponding author on request.
