# Supplementary material for: Experiences of primary care physicians managing postpartum care: a qualitative research study
Source: BMC Fam Pract. 2021 Jun 30;22:139. doi: 10.1186/s12875-021-01494-w (PMC8244666; doi:10.1186/s12875-021-01494-w)
Supplement: Supplementary file 1 — Additional file 1. Interview Guide. [file 12875_2021_1494_MOESM1_ESM.docx]

Supplementary File

| **Supplementary File 1. Interview Guide** |
| --- |
| 1. What do you understand of postpartum care and how long it spans? 2. What are your views regarding the role of primary care physicians in providing postpartum care for women? 3. What is your approach to assess postpartum needs of women? 4. What are the resources that are available for you to better manage these women? 5. How do you think postpartum care can be improved in the primary care sector? 6. What are your views towards telehealth (to show participants a picture of what telehealth consists of) as a new model of care to compliment the current delivery of postpartum care in the primary care setting? |
